# Reminiscences and reflections on kuru, personal and scientific

**DOI:** 10.1098/rstb.2008.0098

**Published:** 2008-11-27

**Authors:** John Collinge, Michael P. Alpers

**Affiliations:** 1 MRC Prion Unit and Department of Neurodegenerative Disease, UCL Institute of Neurology, The National Hospital for Neurology and Neurosurgery, Queen SquareLondon WC1N 3BG, UK; 2 Centre for International Health, ABCRCHealth Research Campus, Shenton Park, Curtin University, GPO Box U1987, Perth, WA 6845, Australia

These reminiscences and reflections are about kuru, its effect on the people who suffered from the disease, the research into kuru over a period of 50 years, and the scientists, doctors and others whose dedicated work led to the solution of this mysterious and devastating disease.

The reminiscences and reflections of 37 participants in the kuru story are gathered here. They are arranged in alphabetical order by author. These historical accounts illuminate the whole period of research on kuru since it began in 1957.

Roy Scragg recalls the early correspondence on kuru and the meeting when he introduced Carleton Gajdusek to kuru. Carleton Gajdusek, Cyril Curtain, J. Henry Bennett, Donald Simpson and Lucy Hamilton Reid provide images and reflections describing kuru and the Fore people, their own memories of Okapa in the late 1950s and the research that they conducted. William Hadlow recalls his seminal recognition in 1959 of the similarity of kuru to scrapie. The responses of the Fore people to the advent of government officers and medical research workers, and the consequent transformation of their traditional life, are briefly described by Taka Gomea, Tiu Pekiyeva, Atenamu Bavasa, Tarubi Taguse and Koiye Tasa. There are memorable accounts from the 1960s by Shirley Lindenbaum, who came as an anthropologist in 1961, Inamba Kivita, who worked with her, Annette Beasley, who recounts Richard Hornabrook's first impressions of kuru and Okapa, and Coralie Mathews, who reflects upon her experiences and those of other ‘kuru wives’. Adolf Saweri and Werner Sto¨cklin were doctors at Okapa Hospital and give a participant observer's perspective on kuru and kuru research. Pako Ombeya became associated with kuru research in 1962 and his son Wandagi Pako describes the work of the current field research project on kuru. Anderson Puwa, Komit Poki, Ken Boone and Jerome Whitfield provide their own perspectives on recent research studies among the Fore and neighbouring people, in which they have all been involved in different ways. Some of the outstanding men who served as research assistants in the field have died; we are privileged to include the tributes of Andemba Anua and Kainamba Mabage to the work of their late husbands. Michael Alpers lays out his tributes to a wide range of colleagues and associates who worked with him on kuru over a period of 46 years. Carleton Gajdusek's laboratory at the National Institutes of Health was the powerhouse of research on kuru for many years and we are fortunate to have reminiscences of life and work in this laboratory from David Asher, Richard Benfante, Françoise Cathala and Judith Farquhar. Of the neuropathologists who made significant contributions to these studies, Byron Kakulas and Gabriele Zu Rhein discuss their introduction to kuru and their engagement with research colleagues. In Papua New Guinea in the 1970s and 1980s kuru studies were vigorously pursued by the Papua New Guinea Institute of Medical Research. Of the colleagues who contributed to the clinical, epidemiological and pathological aspects of this research, Phillip Tarr, Robert Klitzman, Euan Scrimgeour and Stanley Prusiner record their memorable experiences working in the field, intermingled with scientific reflections on kuru.

Further reminiscences and reflections on kuru may be found in the articles by John Mathews (2008) and John Collinge (2008) in this issue.

Among our contributors we are particularly pleased to acknowledge our 2 Nobel Laureates and the 12 Fore men and women who have experienced the bitterness of kuru in their own lives. We join all our colleagues here in celebrating the demise of the kuru epidemic. The collection of their reminiscences and reflections constitutes a remarkable entity spanning the 50 years of research into kuru. Each contribution also has its own individuality, which is marked with a specific identifying doi. Since many of the authors have no email contact they may be reached through either of us at j.collinge@prion.ucl.ac.uk or m.alpers@curtin.edu.au or through our institutional addresses.

## References

[bib1Q1] Mathews J.D. 2008 The changing face of kuru: a personal perspective. Phil. Trans. R. Soc. B 363, 3679–3684 doi:10.1098/rstb.2008.0085 18672465 10.1098/rstb.2008.0085 PMC2581658

[bib2Q1] Collinge J. 2008 Lessons of kuru research: background to recent studies with some personal reflections. Phil. Trans. R. Soc. B 363, 3689–3696 doi:10.1098/rstb.2008.0121 18849283 10.1098/rstb.2008.0121 PMC2577136

